# Erratum

**DOI:** 10.1111/cas.15029

**Published:** 2021-07-02

In an article^1^ titled “Deubiquitinating enzyme inhibitor alleviates cyclin A1‐mediated proteasome inhibitor tolerance in mixed‐lineage leukemia” by Maolin Ge, Qiongyu Xu, Ting Kang, Dan Li, Ruiheng Wang, Zhihong Chen, Shufeng Xie, Wenbin Wang, and Han Liu, the below footnote should have appeared on the first page.

“Maolin Ge and Qiongyu Xu contributed equally to this work.”

The Publisher apologizes for the error.
